# *Ex Vivo* and *In Vitro* Analysis Identify a Detrimental Impact of Neutrophil Extracellular Traps on Eye Structures in Equine Recurrent Uveitis

**DOI:** 10.3389/fimmu.2022.830871

**Published:** 2022-02-10

**Authors:** Leonie Fingerhut, Leyla Yücel, Katrin Strutzberg-Minder, Maren von Köckritz-Blickwede, Bernhard Ohnesorge, Nicole de Buhr

**Affiliations:** ^1^ Department of Biochemistry, University of Veterinary Medicine Hannover, Hannover, Germany; ^2^ Research Center for Emerging Infections and Zoonoses (RIZ), University of Veterinary Medicine Hannover, Hannover, Germany; ^3^ Clinic for Horses, University of Veterinary Medicine Hannover, Hannover, Germany; ^4^ Institute for Animal Breeding and Genetics, University of Veterinary Medicine Hannover, Hannover, Germany; ^5^ IVD Innovative Veterinary Diagnostics (IVD GmbH), Seelze, Germany

**Keywords:** neutrophils, neutrophil extracellular traps (NETs), equine recurrent uveitis (ERU), autoantibody, cytotoxicity, blood-retina barrier

## Abstract

Equine recurrent uveitis (ERU) is a common ocular disease of horses and described as a model for human autoimmune uveitis. This immune-mediated, inflammatory condition progressively destroys the eye, ultimately leading to blindness. Genetic and autoimmune factors, next to infections with *Leptospira*, are discussed as key factors in the pathogenesis. Furthermore, a release of neutrophil extracellular traps (NETs) by activated neutrophils is involved. NETs are composed of decondensed chromatin and proteins that can immobilize invading pathogens. However, if NETs accumulate, they can contribute to detrimental autoimmune processes. Thus, we aimed to investigate the impact of NETs in ERU patients. Therefore, we quantified several NET-markers (cell-free DNA, nucleosomes, citrullinated histone H3, histone-myeloperoxidase complexes, interleukin-17, equine cathelicidin 1 and DNase I activity) and NET-autoantibodies in sera and vitreous body fluids (VBF) of ERU-diseased horses and correlated the data with the disease status (signalment, ERU scores and Leptospira infection status). NET markers were detected to varying degrees in VBF of diseased horses, and partially correlated to disease severity and the presence of *Leptospira* spp. Cell-free DNA and nucleosomes as NET markers correlate with ERU severity in total and VBF scores, despite the presence of active DNases. Additionally, a significant correlation between fundus affection in the eye and NET autoantibodies was detectable. Therefore, we further investigated the influence of VBF samples from equine patients and isolated NETs on the blood-retina barrier in a cell culture model. VBF of diseased horses significantly induced cytotoxicity in retinal pigment epithelial cells. Moreover, partially digested NETs also resulted in cytotoxic effects. In the presence of lipopolysaccharide (LPS), the main component of the leptospiral surface, both undigested and completely digested NETs were cytotoxic. Correlations between the ERU-scores and *Leptospira* were also calculated. Detection of leptospiral DNA, and antibody titers of the serovar Grippotyphosa correlated with disease severity. In addition, a correlation between *Leptospira* and several NET markers was observed in VBF. Altogether, our findings suggest a positive correlation between NET markers with disease severity and involvement of *Leptospira* in the VBF of ERU-diseased horses, as well as a cytotoxic effect of NETs in eyes.

## Introduction

Equine recurrent uveitis (ERU) is a common, inflammatory disease of eyes in horses. Moreover, horses are used as a research model because of numerous similarities in clinical signs and pathogenesis with human autoimmune uveitis (1, 2). In other species, autoimmune uveitis does not occur naturally, and experimentally induced forms are rarely recurrent, in contrast to human and equine disease (1, 3). Additionally, an ERU-like form is inducible in horses by immunization with retinal antigens, and even recurrent after interphotoreceptor retinoid-binding protein injection (4, 5). The spontaneous disease clinically appears with repetitive inflammatory episodes of the eye without systemic findings. Because of the recurrent appearance, the affected eyes are cumulatively destroyed over time, even though uveitic phases are discontinued by subclinical periods. Frequent chronic findings are manifested in the iris, lens, vitreous body and fundus, and can be categorized in a scoring system (6). Typical clinical pictures are synechiae, cataracts, lens luxation, liquefication and inflammatory deposits inside the vitreous, chorioretinopathies up to retinal detachment, and phthisis bulbi (6–9). Therapeutic attempts in ERU patients usually start with local application of immunosuppressive drugs, but in many cases surgical intervention is needed to prevent further painful episodes and finally blindness. One common surgery for horses suffering of ERU is vitrectomy, a minimally invasive replacement of the vitreous body fluid (VBF) by buffered salt solution (8, 10).

Throughout progression of ERU, the permeability of the blood-retina barrier (BRB) increases (11). This enables peripheral immune cells to enter and accumulate in the physiologically immune-privileged compartment (12). However, the cause for this barrier breakdown is unclear, as well as the chronological order of loss of barrier function and cell invasion (13). Nevertheless, multiple factors have been identified that influence the pathogenesis. These include genetic (14) and autoimmune (15–18) components, but also indications for an intraocular infection with *Leptospira* spp. The latter is detectable using culture methods, polymerase chain reaction (PCR), ELISA or microscopic agglutination test (MAT) in about 60 to 100 percent of the affected eyes, but only exceptionally in healthy control eyes (6, 19–22). Systemic *Leptospira* infections with clinical appearance are rare in horses, and the exact role of this pathogen in ERU is under discussion. Direct bacterial effects are suggested as contributing factor or initiating event (6, 23, 24). Persistent infections (23, 24), for instance due to biofilm formation inside the vitreous (25), could explain the recurrent nature. Moreover, autoimmune processes with cross-reactions between leptospiral lipoproteins and ocular proteins were observed, which could also explain the repetitive manifestation (26, 27). Furthermore it has been shown that other autoimmune processes are driven by the dysregulation of immune cells and the complement system (28), as well as the occurrence of autoantibodies (15, 16, 18). In addition, cross-reactivity of antibodies against leptospiral lipoproteins and ocular structures has been reported (27). While the main effects in ERU are attributed to modified lymphocytes (13, 15, 17, 29–31), especially CD4+ T cells, recent ERU studies have also focused in cells of the innate immune system. This is of interest, as horses have a granulocytic hemogram with 45-70% neutrophils among the total leukocyte count (32). Various proteomic changes in granulocytes indicate a latent activation in ERU patients, even during quiescent phases (33). Proteins with enhanced abundance allocate to the pathways of degranulation, antigen presentation and NF-κB signaling. In addition, a cross-presentation of autoantigens by neutrophils was suggested (33). Furthermore, a contribution of neutrophil extracellular traps (NETs) to the pathogenesis of ERU has recently been reported by our group (34).

These trap-like structures are released from neutrophils and can bind pathogens (35). They are composed of decondensed chromatin decorated with hyper-citrullinated histones, granule components and antimicrobial peptides (AMP) (35, 36). Thus, complexes of eight histones wrapped by 147 base pairs of DNA and connected by linker DNA attached to histone 1, known as nucleosomes, are substructures of NETs. One of the adhering granule components is myeloperoxidase (MPO), which is also required for NET formation (37). Furthermore, AMPs protect the DNA backbone against degradation by nucleases (38) and are part of the antimicrobial impact of NETs. The human AMP LL-37, a cathelicidin, is described as a NET inducer (39). However, NET release does not only result in positive effects for the host. NETs can be detrimental towards epithelial and endothelial cells (40–42). Several non-infectious diseases are enhanced by NETs, for instance thrombosis or cancer (43, 44). If NETs accumulate, they may further impede other processes or lead to a more severe manifestation of the disease (45, 46). In this way, NETs enhance autoimmune processes by exposing intracellular structures. For instance, in the course of systemic lupus erythematosus (45), hidradenitis suppurativa (47) and rheumatoid arthritis (48, 49), the production of autoantibodies against NET components occurs. This leads to a protection of NETs against degradation by nucleases (45) and thus to a prolonged presentation of autoantigens. Furthermore, immune complexes can cause disease if they precipitate (50). Antibodies directed against granule proteins of neutrophils, so-called antineutrophil cytoplasm autoantibodies, also induce NET formation in autoimmune small-vessel vasculitis (51).

Our group recently identified more NET markers in sera of ERU patients compared to healthy horses. NETs were furthermore found inside the eyes of affected horses. Additionally, increased NET-release by neutrophils in the presence of VBF of diseased horses compared to healthy horses suggest that factors influencing neutrophils are present inside affected eyes (34).

The aim of this study was to further investigate the correlation of NETs with the disease severity and manifestation of ERU. We analyzed sera and VBF samples of ERU horses and horses with healthy eyes for NET markers and antibodies against NET proteins. In a next step, the corresponding patient records were included in the analysis and correlated with NET markers. Moreover, selected VBF samples were tested for cytotoxicity against cells of the blood-retina barrier. The effect of NET- components against cells of this barrier was investigated using a human cell culture.

## Materials and Methods

### *Ex Vivo* Samples From Humans and Horses

Human blood samples were obtained with the help of a human physician at the University of Veterinary Medicine Hannover, Germany from healthy volunteers. Blood withdrawal from humans for neutrophil isolation was in agreement with the local ethical board. The study was approved by the Ethic Committee of Hannover Medical School (MHH), Hannover, Germany, and registered under no. 3295–2016. The blood collection followed the local guidelines.

All equine samples included in this study were obtained in the Clinic for Horses of the University of Veterinary Medicine Hannover, Germany. Blood collection from horses was approved by the Lower Saxony State Office for Consumer Protection and Food Safety (LAVES) (Niedersächsisches Landesamt für Verbraucherschutz und Lebensmittelsicherheit) under no. 18A302. Fresh equine neutrophils were isolated from these lithium-heparinized blood samples. Sera and VBF from ERU-diseased horses were remnant samples from diagnostic procedures and taken when the patients were at the clinic for vitrectomy. The serum and VBF samples of diseased horses are matched. Additionally, control sera from five healthy horses were obtained. The samples were remnant samples from diagnostic procedures. These control horses had no history of previous ERU episodes. Additionally, the horses were clinically and ophthalmologically examined by an experienced veterinarian and classified as clinically unremarkable with an ERU score of zero. The blood of both, ERU patients and control horses, was collected in serum tubes and left at room temperature until clotting was completed, centrifuged and serum was stored at -80°C until further analysis. VBF samples of ERU patients and healthy control horses were obtained between April 2019 and February 2021 as previously published (34). Inclusion criteria were the availability of complete patient records and results from MAT for intraocular leptospiral antibodies and PCR detecting leptospiral DNA.

### Equine Patient Data

Signalment and clinical data were retrieved from the medical records. For every horse included in this study, breed, gender, and age was collected as general information. Furthermore, specific data about the course of the ERU disease were noted: age at date of vitrectomy, affected side, number of episodes (if available), ERU score and the results of *Leptospira* examinations (MAT and PCR). The ERU score of each eye is comprised of sub-scores ranging from zero (=unaffected) to five (=severely affected) for iris, lens, vitreous body, fundus oculi and other chronic changes, as well as a total score. This system was developed by von Borstel et al. (6) and can be retraced in English in (52) and is summarized in **Table S1**.

The 40 included ERU patients were chosen from a pool of patients whose serum and VBF had been collected between July 2019 and February 2021 at the Clinic for Horses of the University of Veterinary Medicine Hannover, Germany. Of this pool, patients with incomplete data or previous gentamicin injection in the eye were excluded. If samples from both eyes of a horse were available, the side with the higher ERU score was chosen. In case of an equal score on both sides, the *Leptospira* positive side was chosen. This was decided to prevent a possible influence on results of serum examinations by severity of disease or presence of *Leptospira* caused by the more affected eye, while examining the less affected one.

### *Leptospira* Analysis in VBF

All included equine VBF samples were analyzed for *Leptospira* using MAT and PCR.

MAT testing was performed according to the OIE Manual of Diagnostic Test and Vaccines for Terrestrial Animals 2018 (53) using live antigens of *Leptospira* serovars Australis (strain Ballico), Bratislava (strain Jez Bratislava), Autumnalis (strain Akiyami A), Canicola (strain Hond Utrecht IV), Grippotyphosa (strain Moskva V), Copenhageni (strain M20), Icterohaemorrhagiae (strain RGA), Pomona (strain Pomona), Hardjo (strain Hardjoprajitno), Saxkoebing (strain Mus 24), and Tarassovi (strain Perepelitsin). The strains were supplied by the Leptospirosis Reference Laboratory (at KIT Biomedical Research, The Netherlands). Samples were pretested at a final dilution of 1/100. Samples with 50% agglutination were retested to determine an endpoint, using dilutions beginning at 1/25 through 1/3200. Serum samples with the minimum significant titre of 100 (reciprocal of the final dilution of serum with 50% agglutination) were assessed positive.

For PCR, nucleic acid was extracted from VBF using an RNA-DNA isolation kit (MagMAX Pathogen RNA/DNA Kit, Life Technologies GmbH) and an automated nucleic acid isolation processor (MagMAX Express-96 Magnetic Particle Processor, Life Technologies GmbH) that used magnetic bead technology. A total of 700 μL of lysis/binding solution from the kit was added to 300 μL of the sample. Then, 600 μL was transferred on a 96-well plate into the processor, and nucleic acid isolation was performed according to the manufacturer’s protocol and instructions. The PCR of the samples acquired up to and including the year 2020 were analyzed *via* LipL32 real-time PCR according to Ferreira et al. (54). Samples from beginning the year 2021 onwards were analyzed *via* Multiplex real-time PCR according to Perez et al. (55).

### NET Positive Control

Fresh equine neutrophils were isolated from lithium-heparinized blood as published previously (34). In short, neutrophils were isolated using Biocoll (Bio-Sell L6115) density gradient centrifugation and subsequent lysis of erythrocytes with hypotonic sodium chloride.

The NET positive control for the Pico Green assay, the nucleosome ELISA and the histone-MPO ELISA was generated by seeding fresh isolated blood-derived equine neutrophils in a concentration of 2x 10^6^ cells mL^-1^ (2x 10^5^ cells per well). Per well, 100 µL methyl-β-cyclodextrin (CD, final 10 mM, Sigma-Aldrich, C4555) was added. The plate was centrifuged at 370 g for five minutes and thereupon incubated for 170 minutes at 37°C and 5% CO_2_. Per well, 0.005 U micrococcal nuclease (Sigma-Aldrich N5386) was then added for another ten minutes at 37°C, 5% CO_2_. Afterwards, the plate was centrifuged at 370 g for five minutes. Finally, the supernatant was taken off and stored at -20°C until usage in the respective assays.

### Pico Green Assay

The amount of cell-free DNA in serum and VBF, both of horses with healthy eyes and ERU-diseased horses, was evaluated by a Pico Green assay. The analysis was performed according to the protocol of the Quant-iT™ PicoGreen™ dsDNA Assay-Kit (Thermo Fisher P11496) with modifications. In short, PicoGreen was diluted 1:200 in Tris-EDTA buffer solution (TE) and then mixed 1:2 with the sample in a 96 black flat bottom well plate (BRANDplates^®^ 781608) (100µL final volume). A dilution series of the DNA standard of the kit was used for a standard curve. The plate was measured in a TECAN Spark plate reader after a five-minute incubation period at room temperature in the dark.

### Nucleosome ELISA

Serum and VBF samples of horses with healthy eyes and ERU-diseased horses were examined with a Cell Death Detection ELISA^PLUS^ Kit (Sigma 11774425001) in accordance with the manufacturer’s recommendations for evaluating the amount of nucleosomes in those samples.

### Histone-MPO ELISA

A sandwich ELISA for the detection of histone-MPO ELISA was established. The streptavidin-coated microplate of the Cell Death Detection ELISA^PLUS^ Kit (Sigma 11774425001) was coated with 100 µL per well of an anti-histone-biotin antibody (component 1 of the same ELISA Kit) for two hours at room temperature on a microplate shaker at 200 rpm. The antibody was beforehand diluted 1:20 in 1x phosphate-buffered saline (PBS, Sigma-Aldrich P5493-1L, 10× PBS diluted to 1× PBS in distilled water). Then, the wells were washed twice with 100 µL incubation buffer (component 4 of the ELISA Kit) per well and blocked with 100 µL incubation buffer per well for one hour at room temperature while shaking at 200 rpm. 100 µL serum or VBF sample was given into each well and incubated for 1.5 hours at room temperature on a microplate shaker at 300 rpm. A buffer blank was included, as well as isolated equine NET positive control and a custom-made standard as positive controls. This standard was composed of 1.25 µg DNA, isolated from equine neutrophils according to the manufacturer’s recommendations (Thermo Scientific A45721), and 1.25 µg MPO (Recombinant Human Myeloperoxidase Protein, R&D systems 3174-MP), incubated together for 30 minutes at 37°C, 5% CO_2_. This solution was then filled up to a total volume of 100 µL with incubation buffer. After incubation of the samples, the wells were washed three times with PBS-0.05% Tween20 (Roth 9127.2), followed by incubation with 100 µL rabbit anti-human MPO antibody [Merck Millipore #07-496-I, 1 mg/mL; 1:200 diluted in 1% PBS-BSA (bovine serum albumin, Roth CP84.2)] for 1.5 hours at room temperature and 250 rpm. After another three washing steps with PBS-0.05% Tween20, an incubation with goat anti-rabbit IgG HRP conjugated (Merck Millipore #12-348, 1:5000 diluted in PBS) for one hour at room temperature at 250 rpm was performed. 100 µL of TMB ELISA Substrate (High Sensitivity, abcam ab171523) were added for 25 minutes after three washes. The reaction was stopped by adding 100 µL 450 nm Stop Solution for TMB Substrate (abcam ab171529). Readouts were performed by a plate reader (Multiscan Go, Thermo Scientific N13133) at 450 nm minus blank value.

### Citrullinated Histone H3 ELISA

Samples were analyzed with a Citrullinated Histone H3 (Clone 11D3) ELISA Kit (Cayman Chemical 501620) following the manufacturer’s instructions. Serum samples were diluted 1:4, VBF samples 1:2 before measurement.

### IL-17 ELISA

Serum and VBF samples of horses with healthy eyes and ERU-diseased horses were examined with a Horse Interleukin 17 (IL-17) ELISA Kit (Biozol MBS2700989-96) in accordance with the manufacturer’s recommendations for evaluating the amount of IL-17 in those samples.

### Equine Cathelicidin ELISA

Serum and VBF samples of horses with healthy eyes and ERU-diseased horses were examined with a Horse Cathelicidin 1 (CATHL1) ELISA Kit (Biozol MBS9374758-96) in accordance with the manufacturer’s recommendations for evaluating the amount of equine cathelicidin 1 in those samples.

### DNase Activity

DNase I Activity Assay Kit (BioVision, Milpitas, California, USA, Fluorometric, K429-100) was used to determine the DNase I activity in the serum and VBF samples. The test was performed following the manufacturer’s instructions with 25 µL of each sample.

### Quantification of Autoantibodies Against NET Proteins

A Western blot-based detection of autoantibodies against NET proteins in equine patient samples was modified from Carmona-Rivera and Kaplan (56). For this purpose, fresh isolated equine neutrophils were seeded in a concentration of 1x 10^6^ neutrophils per 500 µL in a 24-well plate and 500 µL methyl-β-cyclodextrin (Sigma-Aldrich, C4555) was added to a final concentration of 10 mM. The plate was incubated in a humidified 5% CO_2_ incubator at 37°C for four hours. Afterwards, 10 U/mL micrococcal nuclease was added and incubated for another twenty minutes. After incubation, the liquid from each well was collected in a tube and centrifuged at 300 g for five minutes at 4°C. The resulting supernatant (isolated NETs) was transferred to a new tube and stored at -20°C. Quantification of proteins in the isolated NET samples was conducted using a Bradford assay following the manufacturer’s recommendations (Bio-Rad #500-0006). For the Western blot, 20 µg isolated NETs (protein content) were mixed with 5 µL of sample buffer per well. This buffer was composed of 10% sodium dodecyl sulfate (SDS, Roth CN30.3), 50% glycerine (Roth 3783.1), 250 mM Tris-HCl (Roth 5429.1 diluted in distilled water, pH 6.8) and 0.02% bromophenol blue (Sigma-Aldrich B-6131) in distilled water. Samples were heated at 100°C for five minutes, then put on ice for two minutes and subsequently centrifuged for three minutes at 6200 g. The samples were loaded into the wells of a 4-20% gradient gel, along with a protein ladder (Thermo Scientific #26619) as first band. The gel was blotted with a semi-dry method onto a nitrocellulose membrane (Roth 9200.1). The membrane was then cut into lines along the blotted bands. To enable accurate cutting, the proteins on the membrane were stained with Ponceau S solution (Sigma-Aldrich P7170) for five minutes, cut and then destained using 0.1 M NaOH for one minute. The membrane pieces were subsequently blocked for one hour at room temperature on a shaker in 2.5 mL 10% BSA-PBS per piece. Afterwards, each piece was incubated overnight at 4°C while shaking in 1 mL of different serum or VBF samples. The serum and VBF was therefore diluted 1:250 in 5% BSA-PBS. One identical serum sample was included in each blot to enable quantification of the signals in relation to this standard sample. The following day, each lane was washed five times for five minutes with 3 mL wash buffer [0.1% Tween20 (Roth 9127.2) in Tris buffered saline (20x TBS (Sigma-Aldrich 20-190) diluted to 1x TBS in distilled water)] on an orbital shaker. The secondary antibody (IgG, IgM, IgA (H+L) Rabbit anti-Equine, FITC, Invitrogen™ (Invitrogen SA136092), 1:300 diluted in 5% BSA-PBS) was added for two hours at room temperature while shaking. Then, each lane was again washed five times five minutes with 3 mL wash buffer on an orbital shaker. The readout was performed using a ChemiDoc MP system (Bio-Rad) with fluorescent readout for the serum- or VBF-incubated samples and calorimetric settings for the ladder. Quantification was performed using Image Lab software (Bio Rad, version 5.2.1).

### Cell Culture

A human retinal pigment epithelium cell line (ARPE-19) was obtained from American Type Culture Collection (ATCC, Manassas, VA, USA). The cells were kept in medium 1 [composed of DMEM:F12 (gibco 11330032), 10% fetal calf serum (FCS, Sigma F7524) and 1% penicillin-streptomycin (Sigma P4333)]. For the assays, cells were seeded in a concentration of 4x 10^5^ cells per well in 500 µL medium 1 in a 24-well plate (Greiner Bio-One 662160). For the microscopic evaluation, cells were seeded onto sterile glass slides (12 mm, Roth P231.1). They were kept at 37°C and 5% CO_2_ for 20 hours until confluency was reached. Then, the wells were washed once with phosphate-buffered saline (1x DPBS, gibco 14190144) and 400 µL of medium 2 was added for four hours. Medium 2 was composed of DMEM:F12 and 1% penicillin-streptomycin, without FCS. Cells were subsequently washed with DPBS before 400 µL stimulus was added per well for a period of 20 hours. In all assays, a methanol control was kept in medium 2 for 19.5 hours and then replaced by 70% methanol (Roth 4627.4). A Triton control consisted of medium 2, to which 8 µL 10% Triton [Triton X-100 (Sigma T8787) in RPMI] was added for the last 15 minutes of stimulation. Further controls included were RPMI and medium 2 alone. At the end of incubation, the plate was centrifuged for five minutes at 370 g. Supernatants were taken off for further analysis. The remaining cells were covered with 200 µL of medium 2.

### Equine Patient Samples and Human NETs on Cell Culture

Equine VBF samples were tested for cytotoxic effects towards ARPE-19 cells. Therefore, from the VBF samples included in the NET screening, five with the highest values in the nucleosome ELISA and all five samples from horses with healthy eyes were chosen to be examined. VBF was given onto confluent cell culture cells and incubated for 20 hours at 37°C, 5% CO_2_.

Human neutrophils were isolated from heparinized blood as published (57). Briefly, blood was layered onto a sodium diatrizoate and dextran solution (PolymorphPrep, Progen 1114683) and neutrophils were separated by density gradient centrifugation. Remaining erythrocytes were lysed using sterile water. These fresh isolated neutrophils were seeded in a concentration of 2x 10^5^ cells per well. 100 µL cell suspension were mixed with 100 µL of one of the following stimuli: RPMI, phorbol myristate acetate (PMA, final 25 nM, Sigma 524400), LL-37 (Mobitec AS-61302, final 5 µM). Each of the stimuli was used in nine wells with neutrophils. Five units per well desoxyribonuclease I from bovine pancreas (DNase I; Serva 18535.02, 2000 U/mL) were added to three wells per stimulus. The plate was subsequently centrifuged at 370 g for five minutes at room temperature and incubated for 170 minutes at 37°C and 5% CO_2_. Then, micrococcal nuclease (MN; Sigma-Aldrich N5386) was added to three other wells of each stimulus in a final concentration of 0.5 U/mL and incubated for another ten minutes. The plate was centrifuged (5 min, 370 g) before taking off the supernatants. The triplicate supernatants of each stimulus were carefully taken off, mixed, and immediately added to the cell culture cells. All stimuli were tested on the ARPE-19 cell line with and without addition of LPS (lipopolysaccharides from *Escherichia coli* O55:B5; Sigma L2880) to a final concentration of 10 µg/mL. The stimuli were then incubated with the ARPE-19 cells for 20 hours at 37°C at 5% CO_2_, until readouts were performed.

### LDH Assay

The supernatants of the cell culture experiments were examined to detect the released lactate dehydrogenase (LDH) at the end of the incubation period using the LDH-Glo™ Cytotoxicity Assay (Promega J2380) according to the manufacturer’s recommendations. The VBF samples were additionally measured before incubation, to determine their intrinsic LDH amount.

### Cell Viability Assay

The ARPE-19 cells were tested for viability after incubation with VBF, using the cell counting kit-8 (CCK-8, Sigma 96992) in accordance with the manufacturer’s recommendations. In short, 20 µL of the CCK-8 solution were added to the medium, incubated at 37°C and 5% CO_2_ over 2 hours and measured using a plate reader (TECAN Spark) at 450 nm versus 600 nm. This kit calorimetrically quantifies viable cells through the transformation of WST-8 [2-(2-methoxy-4-nitrophenyl)-3-(4-nitrophenyl)-5-(2,4-disulfophenyl)-2H-tetrazolium, monosodium salt] into formazan by cellular dehydrogenases of living cells.

### Immunofluorescence Staining, Microscopy, and Evaluation of ARPE-19

ARPE-19 cells were carefully washed with 1x DPBS after incubation with VBF samples, to then be fixated with paraformaldehyde (Science Services E15710-250, final concentration of 4%) for 15 minutes at room temperature. This was immediately washed off with 1x PBS (PBS, Sigma-Aldrich P5493-1L, 10× PBS diluted to 1× PBS in distilled water) and plates were stored at 4°C until further investigation. Before staining, the cells were carefully washed again, and then blocking and permeabilization were performed with 0.5% Triton X-100 (Sigma-Aldrich T8787) and 1% bovine serum albumin (BSA; Albumin fraction V, Roth 2923225) in PBS over a period of 60 minutes at room temperature. After washing, Alexa Fluor 546 phalloidin (Thermo Scientific A22283, 200U/mL, 1:40 diluted in 1% BSA-PBS) was added for 45 minutes at room temperature in the dark. Subsequently, cells were washed and a staining with aqueous Hoechst 33342 (Thermo Fisher 62249, 1:1000 in aqua dist.) was conducted for ten minutes. The slides were washed and embedded in ProLong^®^Gold antifade reagent (Invitrogen P36930). A Leica TCS SP5 AOBS confocal inverted-base fluorescence microscope with an HCX PL APO 40× 0.75–1.25 oil immersion objective was used to record the samples. In each sample, six randomly selected images were taken. Respective positive (medium) and negative (methanol) controls were analyzed in parallel. Area and intensity measurements were performed using Image J version 1.51.0.

### Statistical Analysis

Data were analyzed using Excel 2019 (Microsoft) and GraphPad Prism version 9.0.121.0 (GraphPad Software, San Diego, CA, USA). Normal distribution was tested using the Kolmogorov-Smirnov normality test (GraphPad software, San Diego, CA, USA). Differences and relations between groups were investigated as described in the figure legends (*p < 0.05, **p < 0.01, ***p < 0.001, ****p < 0.0001).

## Results

### NET Markers Are Present in Serum and VBF Samples of ERU Patients

**Figure 1:**
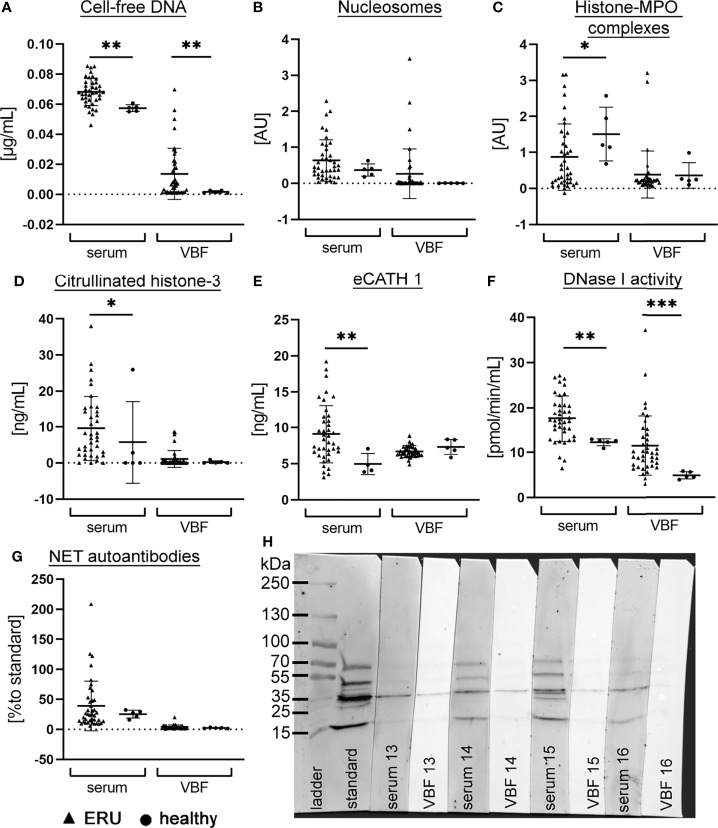
NET markers in serum and vitreous body fluid (VBF) of ERU patients and control horses. The amount of **(A)** cell-free DNA, **(B)** nucleosomes, **(C)** histone-myeloperoxidase (MPO) complexes, **(D)** citrullinated histone-3, **(E)** equine cathelicidin 1, **(F)** DNase I activity and **(G)** NET-autoantibodies was analysed in serum and VBF of 40 horses with equine recurrent uveitis (ERU) and five [four sera in eCATH 1] control horses without signs of ERU. For each marker, the individual values and the mean value ± SD of the group is given. An unpaired one-tailed Mann-Whitney test student’s t-test was calculated (*p <*0.05, **p <0.01 ***p <0.001). Mean values of serum were higher than in VBF, however a wide range is seen in most markers. In **(H)**, a representative image (patient no. 13-16) of a Western blot analysis for the detection of autoantibodies against NET proteins in serum and VBF is depicted. The standard is one identical serum sample that was included in each blot to enable quantification.

To evaluate the extent of NETs in ERU-diseased horses, screening for NET markers in serum and VBF of 40 ERU-diseased horses was performed. Furthermore, serum and VBF of horses with healthy eyes were included in each assay to generate values in non-diseased horses (**Figure 1**). Firstly, we quantified cell-free DNA, nucleosomes and histone-MPO complexes as NET markers. The mean value ± SD of cell-free DNA in serum samples of ERU-diseased horses was 0.068 ± 0.009 µg/mL, whereas their VBF yielded values of 0.014 ± 0.017 µg/mL (**Figure 1A**). Also in nucleosomes, a wide spectrum was observed in ERU samples, with 0.634 ± 0.574 AU in serum and 0.265 ± 0.685 AU in VBF (**Figure 1B**). Complexes of histone and MPO were detected in only very few of the VBF samples (**Figure 1C**). The mean value in serum samples was 0.871 ± 0.923 AU, whereas for VBF 0.387 ± 0.651 AU. An extensive post-translational modification of histone 3, in terms of a citrullination (H3-cit), takes place during NET release. Therefore, we measured the amount of H3-cit in the equine patient samples (**Figure 1D**). The mean value in serum samples was 9.613 ± 8.931 ng/mL. The corresponding VBF samples of ERU patients yielded an H3-cit amount of 1.099 ± 2.336 ng/mL. Additionally, the interleukin IL-17 was measured by ELISA. IL-17 recruits and activates neutrophils (58, 59), and furthermore triggers NET release (59), but is also an indicator for the activity of CD4+ T cells. However, except for three samples, all samples had IL-17 values below the detection limit of 15.6 pg/mL, therefore no further analysis for IL-17 could be conducted. The equine cathelicidin 1 (eCATH1) as a likely part of NETs (60) was measured by ELISA. The mean value of eCATH1 in serum was 9.117 ± 4.009 ng/mL and in VBF 6.698 ± 0.824 ng/mL (**Figure 1E**). The level of eCATH1 in serum was significantly higher in ERU patients than in controls. DNase activity, crucial for the degradation of the NET-backbone, was measured in serum samples with a mean of 17.54 ± 5.090 pmol/min/mL, and in VBF with a mean of 11.49 ± 6.568 pmol/min/mL (**Figure 1F**). The presence of more cell-free DNA and DNase activity in serum of ERU diseased versus healthy horses, as recently reported by our group (34), was confirmed in this set of samples (**Figures 1A, F**). In addition, these two NET markers were also significantly elevated in VBF of ERU patients.

Altogether, these findings support the presence of various NET markers in serum and VBF of ERU-diseased horses. Furthermore, the varying amounts in samples of ERU patients suggest influencing factors, for instance a possible connection to the different stages of disease at the time of sample collection. Since VBF can be collected exclusively during surgery in the quiet phase of ERU, but not in acute inflammatory episodes, we additionally measured autoantibodies against NET proteins in the samples. The presence of autoantibodies inside the eye is detectable for a longer time than the actual presence of NETs, because they are rapidlydegraded by host nucleases (45). Results of autoantibodies against NET proteins in sera were 39.21 ± 41.46% compared to a standard serum sample and in VBF 4.089 ± 3.305% (**Figures 1G, H**).

### Correlation Between NET Markers and Disease Status

**Figure 2:**
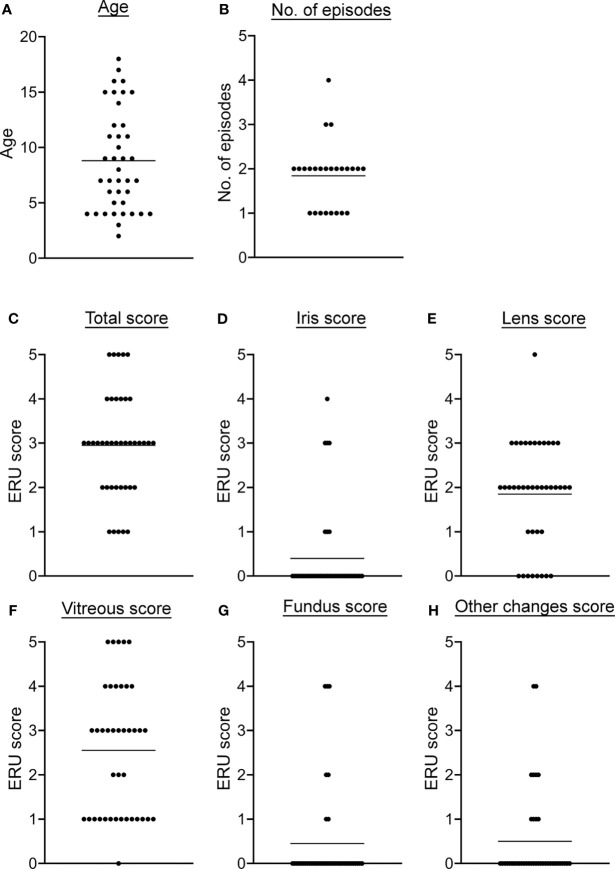
Descriptive data and disease severity classification of ERU patients. **(A)** The age distribution at the examination before the vitrectomy is shown. **(B)** The number of previous ERU episodes of the 25 horses with obtainable data is shown. In **(C–H)**, the total as well as the ERU sub-scores of the different eye structures are given. Individual data points are given along with the mean value for each detail.

After detection of different NET markers in serum and VBF of ERU patients, the main goal of this study was to analyze the correlation with the specific disease status. For this purpose, signalment, clinical data from equine patient records and ocular scores were compared with values of NET markers and autoantibodies against NET proteins. Detailed patient data can be found in the supplements (Tab. S1). Among the 40 examined ERU patients were 60% male horses (23 geldings, 1 stallion) and 40% mares. Their age ranged from two to 18 years, with an average of 8.8 years (**Figure 2A**). The horses included in the study were of various breeds (**Figure S1**). Samples derived from left and right eyes and were equally distributed. Of the included horses, 17 had signs of ERU in both eyes. For 25 horses, owners provided information about the number of previous episodes: One episode had been observed in eight cases, two episodes 14 times, three episodes twice and four in one case (**Figure 2B**). The distribution of the total ERU score showed that most horses were graded between score two and four at the time of surgery (**Figure 2C**). With a proportion of 12.5% each, just minor amounts of horses were slightly (score 1) or most severely affected (score 5). Distributions of the remaining ERU sub-scores are depicted in **Figures 2D–H**. Mean values were below score 1 in iris, fundus, and other changes. Meanwhile, the mean value for the total score was 2.95, for lens 1.85, and in VBF 2.55. All ERU scores are scaled from one to five and thereby express increasing severity of disease.

Neither gender or age, nor the number of previous episodes or the affection of one versus both eyes showed a significant association with disease severity. This was tested with a two-tailed Pearson correlation for data with individual values and by simple logistic regression in case of binary values. The sub-score of the VBF correlated highly with the total ERU score (*p <*0.0001), supporting a detailed investigation of this ocular fluid when examining ERU cases. More correlations could be found when comparing the sub-scores among each other, given the fact that in most ERU patients not only one eye structure is affected.

**Figure 3:**
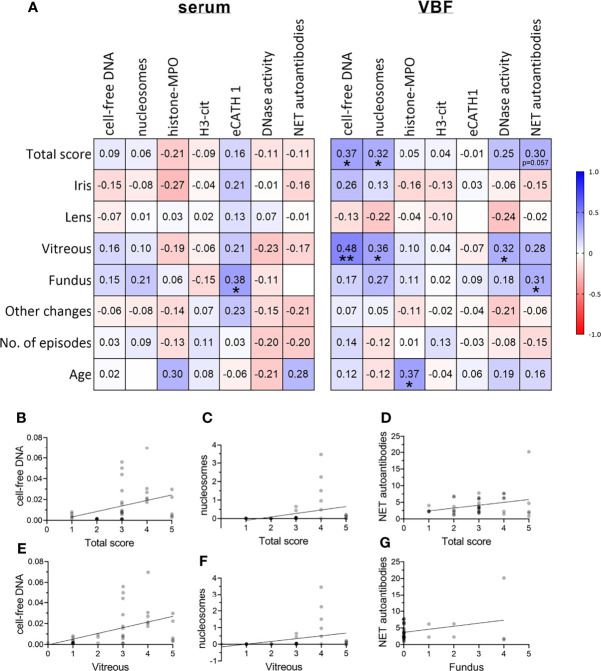
Correlations between severity of ERU and occurrence of NET markers. **(A)** Correlations between ERU scores and NET markers in sera and vitreous body fluids (VBF) of 40 horses with equine recurrent uveitis (ERU) were analysed. Significant correlations between cell-free DNA, nucleosomes, and DNase I activity in VBF with ERU severity were observed. The amount of NET autoantibodies correlated significantly with the fundus score and showed a strong tendency towards the total score (*p*=0.057). A two-tailed Pearson correlation was performed. Pearson’s *r* values are presented, and *p* values are depicted if significant (*p* values of *p <0.05 and **p <0.01 were considered significant). **(B–G)** Scatter plots including the simple linear regression line of significantly correlating NET markers with the total or sub-scores or ERU severity are shown. Individual points are illustrated darker the more data points overlap.

**Figure 4:**
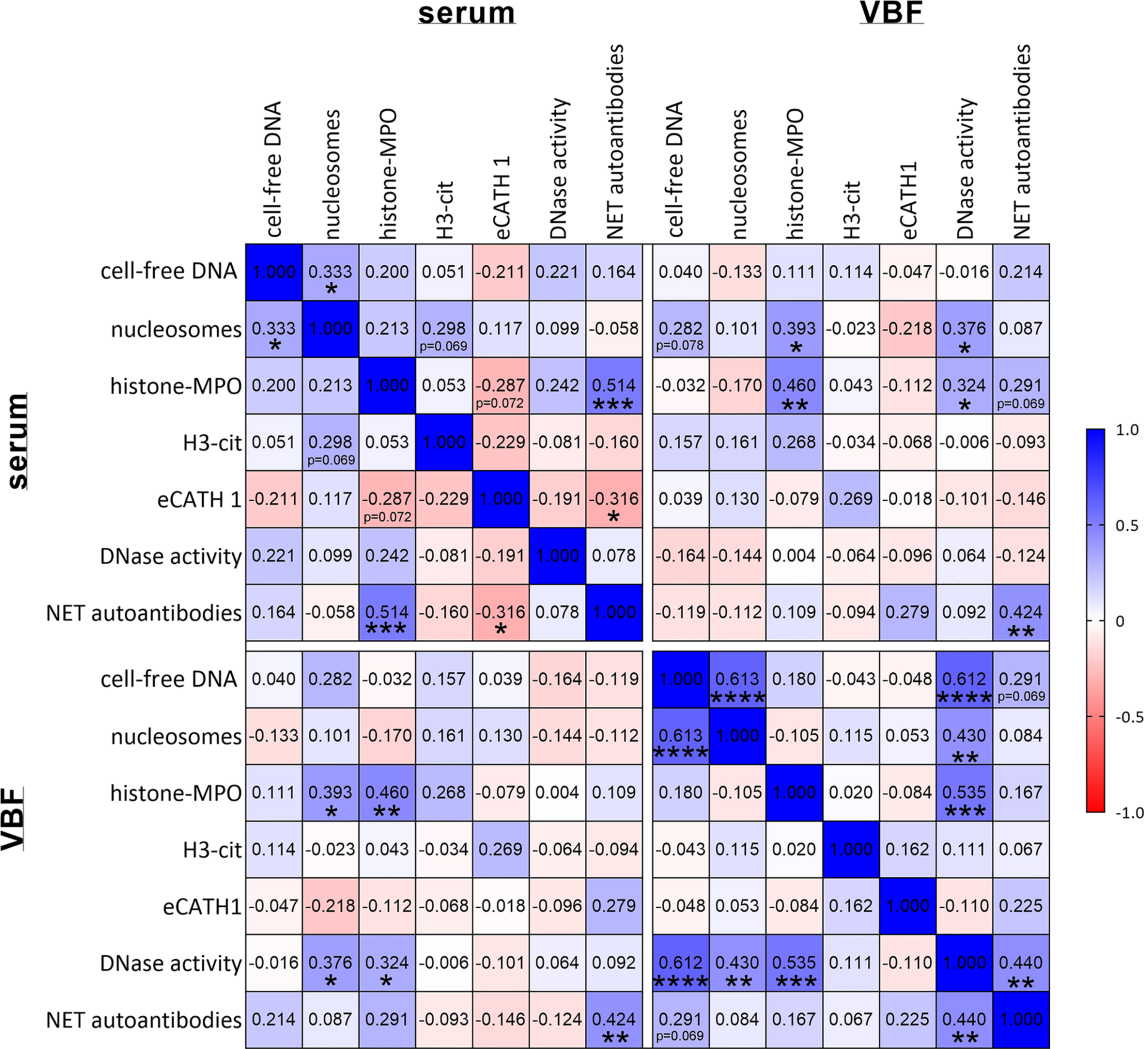
Correlations between NET markers. Correlations between NET markers in sera and vitreous body fluids (VBF) of 40 horses with equine recurrent uveitis (ERU) were analysed. Significant correlations between several NET markers in serum and VBF were observed. The amount of NET autoantibodies correlated significantly with histone-MPO and eCATH1 in serum as well as between serum and VBF. A two-tailed Pearson correlation was performed. Pearson’s *r* values are presented, and *p* values are depicted if significant (*p* values of *p < 0.05, **p < 0.01, ***p < 0.001 and ****p < 0.0001 were considered significant).

The correlations between NET markers in serum and VBF with these clinical data are depicted in **Figure 3A**. No significant link between any of the NET markers and binocular versus unilateral occurrence of ERU, gender, or previous numbers of episodes could be detected. Serum values of NET markers did not correlate with any of the ERU-specific clinical data, except of eCATH1 with the fundus score. However, correlations were observed in VBF samples. Significant correlations exist between cell-free DNA and nucleosomes with the total ERU score and the VBF sub-score (**Figures 3A–G**). This was despite a correlation between the latter score and DNase activity. Histone-MPO complexes, H3-cit and eCATH1 in VBF did not correlate with any of the ERU-specific clinical data. An exception is the correlation between the presence of histone-MPO complexes and the age of the horse (**Figure 3A**). Besides this, a correlation between fundus affection and NET autoantibodies inside the VBF was observed. The amount of NET autoantibodies furthermore showed a strong tendency towards the total score (*p*=0.057). Furthermore, correlations were identified between NET markers in serum and VBF (**Figure 4**). In summary, these results show correlations between numerous NET markers, such as cell-free DNA, nucleosomes, DNase I activity and NET-autoantibodies in VBF of ERU-diseased horses with ERU scores. Hence, these data support a relation between NET occurrence and disease status.

### VBF of ERU-Affected Horses Causes Remodeling and Cytotoxicity in Cells of the Blood-Retina Barrier

**Figure 5:**
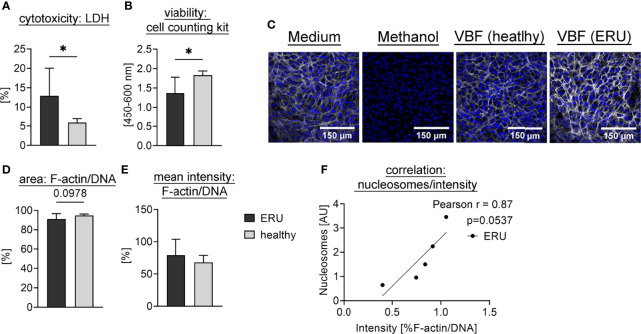
Interaction of equine VBF with cells of the outer blood-retina barrier. Confluent ARPE-19 cells were exposed to VBF of five ERU-diseased and five horses with healthy eyes. For **(A)**, the cytotoxicity was measured using the amount of released LDH. The bars represent the mean ± SD. A one-tailed unpaired student’s t-test comparing healthy to ERU-diseased groups (n=5) revealed a significant difference. *p* values of *p <0.05 were considered significant. **(B)** shows the viability of cells determined by cell counting kit-8. The bars represent the mean ± SD. Viability was significantly higher after incubation with healthy VBF samples compared to ERU samples. A one-tailed unpaired student’s t-test was performed. *p* values of *p <0.05 were considered significant. In **(C)**, representative images of the cells after incubation with medium (DMEM:F12, 1% Penicillin-Streptomycin), methanol (final 70%), VBF of a horse with healthy eyes and VBF derived from an ERU patient are depicted. Confocal microscopy was performed after immunofluorescence staining [blue = DNA, white = F-actin]. In **(D)**, the area covered by F-actin in relation to nuclei was compared between the groups, whereby no difference could be observed. In **(E)**, an increase in the ratio of F-actin to DNA in RPE cells incubated with VBF of ERU patients was detected. For **(D, E)**, six randomly selected images per sample (n=5 per group) were evaluated. The bars represent the mean ± SD. One-tailed unpaired student’s t-tests were performed. In **(F)**, the nucleosome values of the ERU samples show an almost significant correlation to the according intensity ratio. A two-tailed Pearson correlation was performed, and the scatter plot including the simple linear regression line is presented.

To investigate a potential detrimental effect of NET markers in VBF we further tested the effect of equine VBF towards the outer blood-retina barrier in a model using the retinal pigment epithelium (RPE) cell line ARPE-19. For this purpose, VBF of five ERU-diseased horses was compared to five healthy controls. In order to investigate cytotoxicity of NETs, we chose to examine the VBF samples with the highest values regarding nucleosomes. Significantly higher cytotoxicity was measured *via* LDH release after incubation with VBF from ERU patients compared to healthy controls (**Figure 5A**). However, as the VBF samples contain endogenous LDH, even before incubation on ARPE-19 cells (**Figure S2**), the analysis was verified by measuring the viability of cells. For this purpose, the activity of dehydrogenases was quantified, resulting in significantly elevated values after incubation with samples from healthy compared to ERU-diseased horses (**Figure 5B**). Microscopy images after incubation of confluent ARPE-19 cells with VBF samples were obtained after immunofluorescence staining for F-actin and DNA to visualize the condition of the cells (**Figure 5C** and **Figure S3**). The effect on the thickness and intensityof F-actin fibers in cells incubated with VBF of ERU versus healthy controls was investigated in microscopic images by comparing the area covered by F-actin in relation to the DNA of the nuclei (**Figure 5D**). Thereby, no significant differences were detected. Regarding the intensity, with mean values ± SD of 79.14 ± 24.61% versus 67.95 ± 10.92%, a higher but not significant increase after incubation with VBF from ERU patients could be shown (**Figure 5E**). In summary, these data suggest a remodeling and cytotoxic effect of VBF from ERU patients towards retinal pigment epithelial cells of the blood-retina barrier. Moreover, an almost significant correlation between the nucleosome values and the intensity measurement data suggests an involvement of structures containing DNA and histones (**Figure 5F**).

### NETs Are Cytotoxic Towards the Blood-Retina Barrier

**Figure 6:**
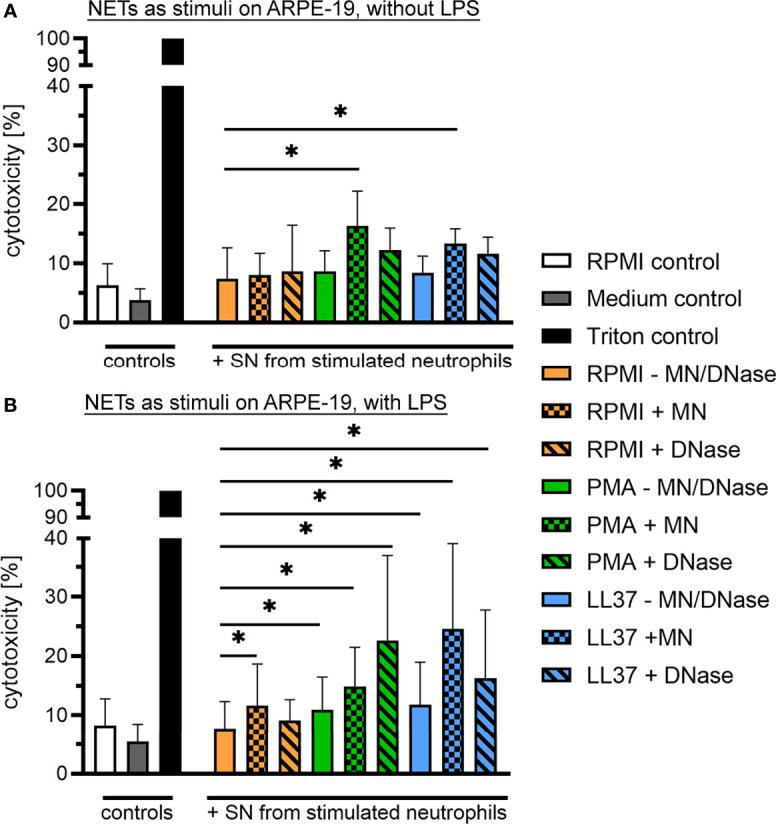
Cytotoxic effect of human NETs on retinal pigment epithelium (RPE) cells. **(A)** Confluent ARPE-19 cells were exposed to supernatants of unstimulated human neutrophils (PMN) or NETs, induced by either phorbol myristate acetate (PMA) or the human cathelicidin LL-37. All variants were tested undigested, partially (micrococcal nuclease, MN) and completely (DNase I) degraded (n=6). Cytotoxicity was measured *via* the release of lactate dehydrogenase (LDH) by ARPE-19 cells. The bars represent mean ± SD. A one-tailed Wilcoxon matched-pairs signed rank test was performed. *p* values of *p <0.05 were considered significant, comparing each stimulus with the neutrophils in RPMI setup. A significantly higher cytotoxicity towards ARPE-19 cells was measured after exposition to MN-digested NETs, induced by either PMA or LL-37 (*p <*0.05). **(B)** Furthermore, the cytotoxicity of all stimuli was evaluated in presence of lipopolysaccharide (LPS; n=6). The bars represent mean ± SD. A one-tailed Wilcoxon matched-pairs signed rank test was performed. *p* values of *p <0.05 were considered significant, comparing each stimulus with the neutrophils in RPMI setup. A significantly higher cytotoxicity towards ARPE-19 cells was measured after exposition to undigested, MN-digested and DNase I-digested NETs in presence of LPS, induced by either PMA or LL-37 (*p <*0.05). In addition, a significant higher LDH-release was measured after incubation with supernatants of neutrophils incubated with MN.

In the VBF, in addition to cell-free DNA or other NET markers, a variety of substances are present that might also be cytotoxic or, conversely, might prevent effects of NETs. Therefore, we further investigated the effect of isolated human NETs towards a human cell line representing cells of the blood-retina barrier. Recurrent uveitis is found in horses and humans with similar pathogenesis, implying a possibility for species extrapolation of results (1, 2). Confluent RPE cells were exposed to supernatants of unstimulated human neutrophils or NETs, induced by either PMA or the human cathelicidin LL-37. These variants of NETs were tested undigested, partially digested by micrococcal nuclease (MN), and completely degraded through DNase I. The release of LDH by dying cells was used to quantify cytotoxicity. A significant increase was observed if cells were exposed to partially, MN-digested NETs, independent of the NET-inducing stimulus (**Figure 6A**). A considerable, inherent cytotoxicity of the NET-inducing stimuli was excluded by incubation of an ARPE-19 cell layer with the stimuli alone (**Figure S4A**). Subsequently, the cytotoxicity of NETs was also investigated in the presence of LPS, an important component of the bacterial surface of the Gram-negative bacteria *Leptospira* spp., which are frequently detected in ERU-affected eyes. Under the influence of LPS, a cytotoxic effect of partially digested NETs was likewise observed (**Figure 6B**). Moreover, a significantly higher cytotoxicity towards ARPE-19 cells was also detected after exposition to undigested and DNase I-digested NETs, compared to supernatants of unstimulated neutrophils. In addition, a significant increase in LDH-release was measured after incubation with neutrophil supernatant incubated with MN, compared to values derived under absence of MN. Overall, these results indicate a cytotoxic effect of human NETs in their degraded occurrence, which is reinforced in the presence of LPS. Moreover, LPS and NETs in combination act detrimental to the cells of the blood-retina barrier, independent of digestion.

### Correlations of *Leptospira* With ERU Severity and NET Markers

**Figure 7:**
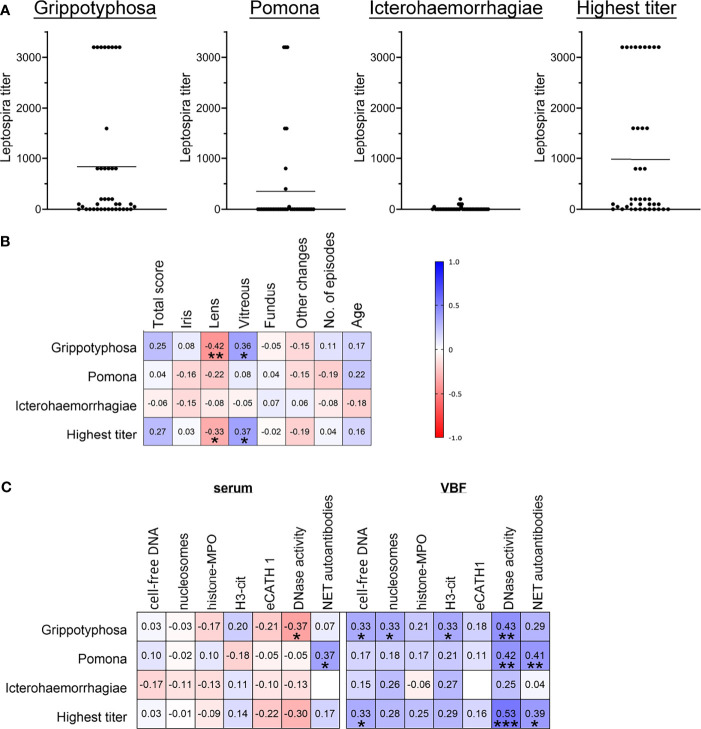
Correlations between severity of ERU, occurrence of NET markers and *Leptospira*. Correlations between ERU scores, *Leptospira* spp. titers and NET markers in sera and vitreous body fluids (VBF) of 40 horses with equine recurrent uveitis (ERU) were tested. Part **(A)** shows the distribution of the three *Leptospira* serovars Grippotyphosa, Pomona and Icterohaemorrhagiae, and of the highest titer value per sample. Individual data points are given along with the mean value for each graph. In **(B)**, the resulting correlation matrix between ERU severity data and *Leptospira* titers is depicted. A significant correlation was found between the vitreous score and serovar Grippotyphosa. Correlations between NET markers and *Leptospira* titers are displayed in **(C)**. Significant correlations between *Leptospira* spp. serovars and various NET markers in serum (DNase I activity, NET autoantibodies) and in VBF (free DNA, nucleosomes, H3-cit, DNase I activity, NET autoantibodies) were observed. A two-tailed Pearson correlation was performed for **(B, C)**. Pearson’s *r* values are presented, and *p* values are shown if significant. *p* values of *p < 0.05, **p < 0.01 and ***p < 0.001 were considered significant.

As LPS reinforced and broadened the cytotoxic effect of human NETs in the cells of the outer blood-retina barrier, we next intended to evaluate if correlations between leptospiral involvement and ERU severity, as well as to the presence of NET markers are detectable for horses. Seventeen of the examined equine VBF samples were positive for intraocular antibodies against *Leptospira* spp. in MAT and in PCR detecting leptospiral DNA, while ten samples were negative in both tests. Only MAT was positive in twelve VBFs, solely PCR in one case. In this study, samples were considered positive for *Leptospira* spp. once one of the test results was positive. The relation between positive or negative values and the total ERU score, representing the severity of disease, was investigated. No link could be observed by the occurrence of this pathogen in MAT, whereas a significant one was seen between PCR results and the total ERU score (Wald test *; Likelihood ratio test *). Moreover, *Leptospira* results were divided into groups according to the detected serovar and graded by titer values (**Figure 7A**). Serovar Grippotyphosa, Pomona and Icterohaemorrhagiae were found in more than one VBF sample, but no correlation to the total score of the disease could be observed (**Figure 7B**). Interestingly, the ERU sub-score of the vitreous and the titers of the serovar Grippotyphosa correlated positively with one another (**Figure 7B**).

Considering the values of NET markers in sera in association with the *Leptospira* infection status (**Figure 7C**), correlations were observed between DNase I activity and the Grippotyphosa titers. Furthermore, NET-autoantibodies in sera correlated with the Pomona titers. Between NET markers in the investigated VBF and *Leptospira* (**Figure 7C**), correlations were noticed for cell-free DNA with both, Grippotyphosa and the highest titer. The cell-free DNA additionally correlated with PCR results of leptospiral DNA. Moreover, the quantity of nucleosomes and H3-cit in VBF correlated with the Grippotyphosa titer. DNase activity in VBF samples was furthermore significantly associated with Grippotyphosa and Pomona titers, as well as with the highest titer. Finally, NET autoantibodies and both, Pomona and highest titers, significantly correlated with each other. In summary, these results show correlations between *Leptospira* and disease severity in ERU patients on one side, but also between various NET markers and leptospiral occurrence especially at the location of the disease.

## Discussion

Nowadays, there is increasing evidence about the role of neutrophils and NETs in the pathogenesis of ERU. Besides a latent activation of these immune cells even in the quiescent state of disease (33), also NETs were found in eyes of ERU patients by our group (34). However, the exact role of this immune mechanism remained unclear. NETs are protective in various infectious diseases but can induce detrimental effects as shown in several autoimmune diseases. The pathogenesis of ERU includes immune-mediated components, as well as indications for a bacterial involvement of *Leptospira* in about 60-100 percent of the cases (6, 19–22) and 75 percent in this study (**Figure 7** and **Table S2**).

Hence, we investigated the occurrence of NETs and their potential link to the disease status in 40 ERU patients. Therefore, sera and VBF samples were screened using various techniques of NET detection. Our results demonstrate the presence of NET markers in varying amounts in both, serum and VBF samples or ERU patients (**Figure 1**): We found cell-free DNA, nucleosomes, histone-MPO complexes, H3-cit, and eCATH1 as NET components, and furthermore DNase I activity and autoantibodies against NET components (**Figure 1**). All these markers indicate an appearance of NETs within the eyes of ERU patients. Importantly, some markers significantly correlated with data describing the disease status. In particular, significant correlations of cell-free DNA and nucleosomes to the total ERU score and the vitreous sub-score exist. At the same time, DNase I activity correlated with vitreous score and confirmed previous data that stabilized NETs may occur and accumulate during ERU disease (34). This points towards an association of NET-formation with disease severity since their backbone is formed by DNA in conjunction with histones (35). Moreover, nucleosomes are accepted indicators of chromatin decondensation and fragmentation (61). However, an origin of the cell-free DNA or nucleosomes other than NETs cannot be excluded. DNA may derive from apoptotic or necrotic neutrophils, or even cells besides granulocytes (62, 63). For instance, a release of extracellular traps by distinct immune cells, such as mast cells (64), macrophages (65), B cells (66, 67), or T cells (66, 68, 69), is possible. Especially T cells are involved in ERU progression (29, 70). Besides immune cells also destroyed tissue could be a source of cell-free DNA. Therefore, we determined further NET markers, which are more specific for these structures derived from neutrophils. Using a histone-MPO ELISA, we aimed for a combination of components in a complex unique for extracellular traps. MPO is an enzyme mainly stored and released from neutrophil granules, but also by monocytes and macrophages. The widely used DNA-MPO ELISA for quantification of NETs was criticized due to the possible formation of such complexes in the extracellular space by electrostatic interaction upon cell death and degranulation (71). In contrast, MPO and histones are both positively charged, and furthermore histones are more specific for the detection of chromatin released by cells than just cell-free DNA. Yet, histone-MPO complexes were scarce in our samples and did not correlate to the patient data, except age at vitrectomy. MPO seems to be quite variable in horses, as reported for the MPO index reflecting the mean neutrophil MPO staining (72). Further assays intended the determination of H3-cit and eCATH1 as NET components (**Figures 1D, E**). The citrullination of histone 3 occurs during the formation of NETs, enabling the decondensation of chromatin inside the nucleus before mixing with granule proteins and expulsion (73, 74). However, no correlations of both markers, H3-cit and eCATH1, with disease status were observed in our study (**Figure 3**). An explanation could be that the gene for eCATH1 is not functional in half of the equine population and the occurrence of the functional peptide *in vivo* is questionable (75, 76).

While the lack of correlations in the latter assays implies that the correlations seen for cell-free DNA and nucleosomes are caused by other sources than NETs, further assays revised this opinion: Autoantibodies against NET proteins were detected and quantified using Western blot (**Figures 1G, H**), and, importantly, their appearance significantly correlated with the condition of the ocular fundus (**Figure 3**). In addition, a strong tendency towards a correlation was observed with the total ERU score (*p*=0.057). Together, this means that more autoantibodies against NET proteins were detectable if eyes were more severely affected. As autoantibodies are detectable for a longer time than NETs, which are rapidly degraded over time by nucleases (45), they are a good indicator of previous overwhelming or impaired NET release. This is especially important considering the DNase activity in our samples, which correlates with increased quantities of NET-markers e.g., cell-free DNA and nucleosomes, indicating that NETs may be stabilized against degradation by nucleases during ERU disease (34). Also autoantibodies are described to contribute to NET stabilization (45).

In particular, the correlation of NET-autoantibodies with the fundus score is meaningful, as the retina is evaluated in this score. Autoantibodies against retinal antigens have already been described in ERU, including intermolecular epitope spreading (15, 77). We have not investigated if such a cross-reaction of NET antigens with other ERU-related antigens exist. Yet, the uveitogenic potential of yeast histone H3, along with its cross-reactivity to the retinal autoantigen S-antigen, has already been shown in rats (78). This indicates potential mimicry between NET antigens and retinal antigens in ERU.

In addition, we examined the effect of equine VBF samples and isolated human NETs towards cells that are part of the retina creating the outer blood-retina barrier. This barrier is composed of retinal pigment epithelial cells, adjacent to Bruch’s membrane (79). It is indispensable for the preservation of the ocular immune privilege, has immunomodulatory functions and furthermore nourishes and protects retinal cells (12, 80). This is crucial, as the equine retina is widely avascular (81, 82). In ERU, the barrier is affected and finally breaks down, resulting in a less controlled in- and efflux of macromolecules and even immune cells into the eye. This access for inflammatory cells through the RPE to the retina was shown in spontaneous as well as in experimentally induced equine uveitis (4, 5, 81). However, it is unclear what causes this barrier breakdown. With our samples, we observed a significant cytotoxicity of VBF from ERU-diseased patients with high level of NET-marker nucleosomes, along with a hint of a remodeling effect towards F-actin (**Figure 5**). An increase of intensity has been observed in immunofluorescence stainings of the actin cytoskeleton, which could be due to the appearance of stress fibers or an event preceding apoptosis. An accumulation of F-actin aggregates takes place when actin turnover is decreased, and regulates cell death (83). A remodeling of the RPE cytoskeleton with stress fiber formation and in association with cell-cell dissociation can be triggered by oxidative stress (84). An almost significant correlation (*p*=0.054) of nucleosome values and actin intensity points towards an involvement of chromatin substructures in the process of remodeling, e.g., as inducing component (**Figure 5F**).

To further determine if NETs could be responsible for the cytotoxic effects of the VBF, we next exposed RPE cells to isolated NETs. That way, further cytotoxic or inhibitory factors present in VBF were excluded. For instance vitreous, and in particular hyaluronan, can neutralize cytotoxic effects of histones (85). Determination of the sole effect of NETs resulted in cytotoxicity of partially MN-digested NETs (**Figure 6**). MN only degrades the linker DNA between nucleosomes but does not affect DNA which is directly bound to histones. This is in contrast to DNase I, an enzyme digesting also DNA-histone complexes (86). A synergistic influence of DNA presence on the action of histones was proposed by Tsourouktsoglou et al. In their study, they report the potentiation of a pro-inflammatory, but sub-lethal, signal of histones towards monocytes through the interaction with DNA in NETs. The cytokine release induction in monocytes was dependent on the chromatin fragment size, and not observable if DNA was completely removed from histones by DNase I (86). However, this observation was regarding sub-lethal doses, and did not address cytotoxicity. Regarding cytotoxicity of NETs, Saffarzadeh et al. proposed a general cytotoxic effect, as they observed a dose-dependent reduction in viability of epithelial and endothelial cells. This was furthermore independent of DNA digestion by MN or DNase. Moreover, the inhibition of histone or MPO resulted in reduced cytotoxicity. Thus, they concluded that the protein content of NETs is mainly responsible for the toxic effects, especially the histones (40). This outstanding role of histones or protein contents for NET cytotoxicity was endorsed in studies evaluating NETs in glomerulonephritis (87) or lung epithelial injuries (42). Such a detrimental effect of extracellular histones was further reported for endothelial cells *in vitro* (88, 89), as well as in mice *in vivo* (88). However, no cytotoxic effects of histones was found once they are part of nucleosomes, but indeed after nucleosome degradation (89). This is in accordance with a study by Marsman et al., where nucleosomes and artificial histone-DNA complexes were only cytotoxic towards human embryonic kidney cells after complete DNA digestion (90). Moreover, injection of nucleosomes did not lead to death in mice (91), in contrast to the injection of free histones (88). This difference to the findings of Saffarzadeh et al., who reported cytotoxicity of NETs degraded into nucleosome-like pieces by MN, could be explained by the chromatin structure. It is more open in NETs and products of NET degradation if compared to nucleosomes of other sources (92), due to modifications of histones in NETs like citrullination. That way, histones can probably still act cytotoxic when bound to DNA in NETs, as they are more exposed than in the tight DNA-histone interaction of nucleosomes. Thus, DNA in nucleosomes might limit histone cytotoxicity by neutralization of the positive charge (90), whereas DNA in NETs might have no influence on histone cytotoxicity. Instead, DNA has here an even enhancing effect by blocking histone degradation through serine proteases (40). On the other side, a protective effect of histones against degradation of extracellular DNA was also suggested (93). Additionally, other components of NETs besides DNA and histones contribute to cytotoxicity, which are not present in nucleosomes, and the length of the chromatin could have another influence (92). However, it often remains difficult to distinguish between cytotoxic effects of free histones, nucleosomes, and NETs in their complete or degraded forms, as no specific antibodies for free versus bound histones exist and often no discrimination is made in the literature (92, 94).

As the majority of ERU patients are positive for *Leptospira*, we also included LPS in our cell culture model. LPS is the major antigen of the leptospiral surface, used to define the serovars and important for immune reactivity (95). In our model, not only MN-degraded NETs were cytotoxic against RPE in presence of LPS, but all variants of undigested and digested NETs. This included NETs induced by the human cathelicidin LL-37, even though LL-37 is capable of binding and modulating LPS signalling, and therefore is strongly antiendotoxic (96). Moreover, a significant increase of cytotoxicity was evoked by supernatants of unstimulated neutrophils with MN. Further studies are needed to understand and explain this observation more in detail. Altogether, these findings suggest a cytotoxic effect of NETs towards the outer blood-retina barrier, especially in the presence of LPS. This enhancing effect of LPS could be explained by the inflammatory response in ARPE-19 cells that is induced by LPS in the used concentration of 10 µg/mL (97). LPS has a lethal concentration LC_50_ to RPE of 17.7 µg/mL (98). The inflammation by lower concentrations is mediated through expression of the primary LPS receptor and its co-receptor by RPE, and leads to enhanced nitric oxygen production, reduced mitochondrial function, increased expression of cyclooxygenase COX-2 and prostaglandin E2, altogether indicating inflammation (97). However, no LDH leakage from ARPE-19 is induced by LPS alone in this concentration, even after incubation for up to 72 hours (97). Such a pro-inflammatory but non-cytotoxic stimulation of ARPE-19 or native RPE cells by LPS, expressed *via* enhanced cytokine release and changes in cytokine receptor expression, was also observed by others, even in human uveitis (98, 99). On the other side, LPS also stimulates autophagy and thereby diminishes its induction of cell death in RPE cells (100). Moreover, LPS induces ADP-ribosylation of histones, which destabilizes their binding to DNA, and thereby promotes accessibility to nucleases (101). This is an intracellular pathway described in macrophages; thus, it remains unclear if this could also occur in the interaction of LPS with NETs and further studies are needed. Despite all these influences of LPS on RPE, it should be mentioned that the bacterial origin of LPS may influence the outcome of its actions, as it has been shown for LPS as NET inducer (102). In addition, in cases of *Staphylococcus aureus, Bacillus cereus* and *Enterococcus faecalis*, live bacteria and their culture supernatants had stronger toxic effects on the retina than inactive bacteria or merely their cell wall. Thus, it was concluded that the retinal damage through - at least Gram positive - bacteria is provoked to a higher extent by secreted toxins than by cell wall components (103). Therefore, to reinforce our findings, the effects of NETs in combination with LPS should be verified using live *Leptospira*. Different serovars that are detected in ERU should be compared using various infection dose and time-points. Additionally, living *Leptospira* could influence the effect of NETs, as two of their outer membrane proteins are capable of inhibiting MPO activity (104). Moreover, pathogenic strains of *Leptospira* can degrade NETs (105) because several surface exposed lipoproteins of *Leptospira* have NET-degrading nuclease activity (106). However, as the cultivation of *Leptospira* and the following *in vitro* experiments with neutrophils and cell culture models are challenging, future studies will focus on this.

As 75 percent of our ERU-affected VBF samples were positive for *Leptospira* (**Figure 2** and **Table S2**), and furthermore correlations between leptospiral occurrence and disease severity were observed (**Figure 7B**), we also examined if there is a link between NETs and *Leptospira* (**Figure 7C**). This hypothesis was supported by the reinforced detrimental effect of NETs towards the blood-retina barrier in presence of LPS (**Figure 6B**). Such correlations were indeed observed for cell-free DNA, nucleosomes, H3-cit, DNase I activity and autoantibodies against NET proteins. Most correlations were found with the titer of the serovar Grippotyphosa (**Figure 7C**). This serovar has a predominant involvement in ERU cases in Europe (107, 108). Beyond this relationship between NET occurrence in ERU samples and *Leptospira*, a NET inducive effect of pathogenic *Leptospira* serovars has already been reported in humans, mice and cattle (105, 109). A limitation of this study in this regard is the lack of *Leptospira* isolation from VBF samples to confirm their current presence at the time of sample acquisition.

Overall, our data suggest a detrimental effect of NETs in ERU, as correlations to disease severity exist (**Figure 3**) and furthermore NETs were cytotoxic towards RPE cells *in vitro* (**Figure 5**). Such a detrimental influence of NETs has already been described for other chronic, autoimmune, or immune-mediated diseases. Extracellular traps (ETs) exacerbate chronic inflammation, for instance because histones impair the removal of dead neutrophils (110). A prolonged exposure of NET components promotes autoimmune reactions, as in systemic lupus erythematosus (45, 111), antiphospholipid syndrome (112) or rheumatoid arthritis (48, 113). It is possible that NETs accumulate due to DNase inhibitors or protection against degradation by autoantibodies (45, 112). In our study, we observed a positive correlation between NET-autoantibodies and DNase as well as between cell-free DNA or nucleosomes and DNase, which could be explained by an inhibition of DNase-mediated NET-degradation in presence of autoantibodies. However, it cannot be excluded that the assays investigating NET occurrence by markers coincidently detected ETs released by other types of immune cells. Therefore, further research determining cell type specific ET components is needed. In addition, the role of mononuclear cells as origin of ETs in ERU should be investigated in future studies.

**Figure 8:**
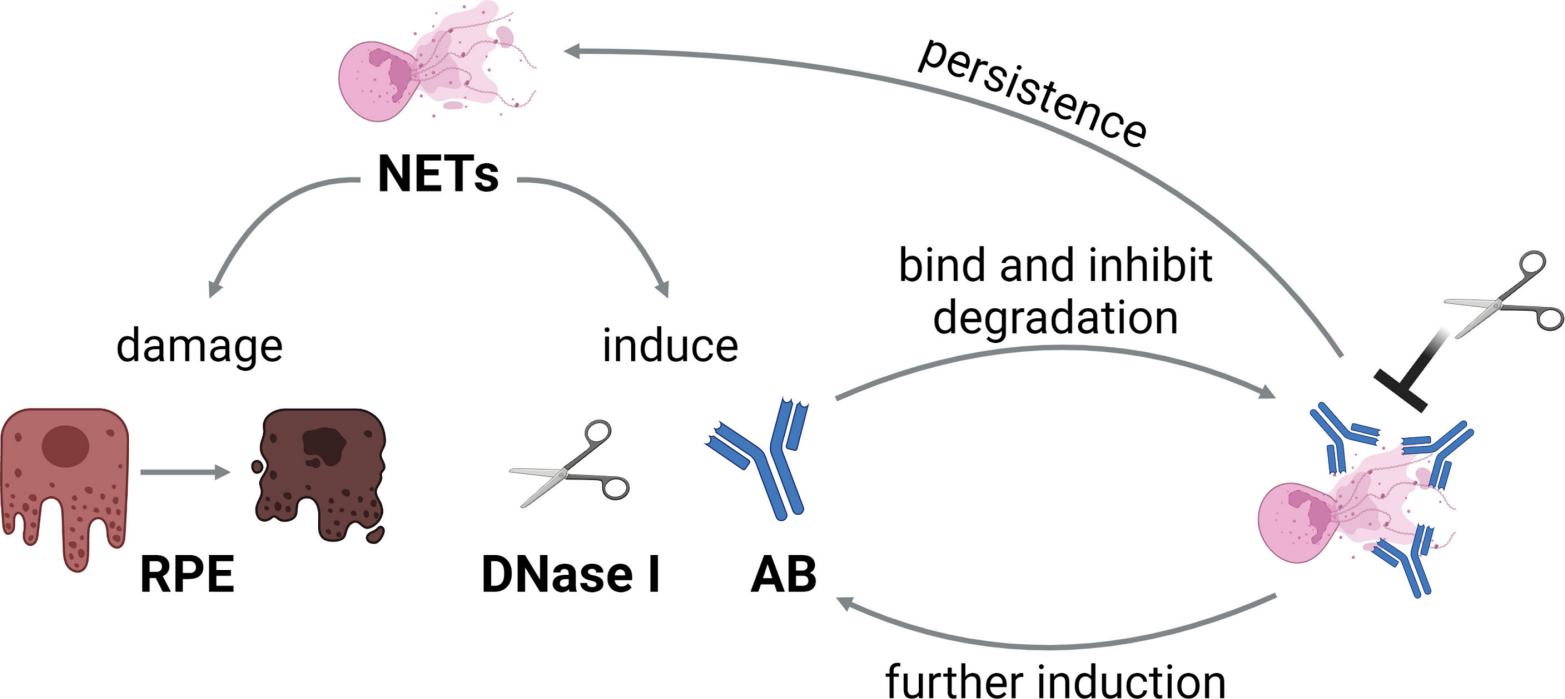
Hypothesis of the vicious circle of NETs and autoantibodies in ERU. NETs induce DNase I and autoantibody production, and furthermore damage the retinal pigment epithelium (RPE). As the autoantibodies bind to NETs, degradation by DNases is impeded. This leads to persistence of NETs, and therefore further induction of autoantibodies, resulting in a vicious circle. The graphic was constructed with BioRender.com.

In summary, we hypothesize that the presence of undegraded NETs indeed induces DNases, however as this enzyme is ineffective in presence of autoantibodies, the NETs remain undegraded. This leads to a positive correlation between those values. Such an effect would result in a vicious circle of NETs inducing autoantibody production, the latter impeding the degradation of NETs, and therefore further induction of autoantibodies by the maintained NETs can occur (**Figure 8**). Since NETs act cytotoxic to retinal epithelial cells, the retinal barrier is damaged and inflammatory processes enhanced that contribute to long-term damage. The cytotoxicity of NETs towards the outer blood-retina barrier in general is interesting, especially as this barrier is involved in multiple eye diseases. Even though the cytotoxicity was not extremely high in absence of LPS, it should be noted that ARPE-19 cells are highly resilient to apoptosis (114). This is in accordance with the *in vivo* situation, as RPE cells need to be robust in order to protect the neural retina (97, 115). Even small changes in RPE function or viability signify immense influences on the photoreceptors and can easily result in loss of vision (97). As the horse is an excellent model for the pathogenesis of human autoimmune uveitis, also detrimental effects of extracellular chromatin and possibly NETs in human eyes should be further investigated in the future.

## Data Availability Statement

The original contributions presented in the study are included in the article/**Supplementary Material**. Further inquiries can be directed to the corresponding authors.

## Ethics Statement

The studies involving human participants were reviewed and approved by Ethic Committee of Hannover Medical School (MHH), Hannover, Germany. The patients/participants provided their written informed consent to participate in this study. The animal study was reviewed and approved by Lower Saxony State Office for Consumer Protection and Food Safety (LAVES) (Niedersächsisches Landesamt für Verbraucherschutz und Lebensmittelsicherheit), Germany. Written informed consent was obtained from the owners for the participation of their animals in this study.

## Author Contributions

Conceptualization, NB, MvK-B, and BO. Methodology, NB, MvK-B, and BO. Validation, NB, LF, and BO. Formal analysis, NB, MvK-B, and LF. Investigation, NB, LF, LY, and KS-M. Resources, BO, NB, and MvK-B. Data curation, NB, LF, and BO. Writing—original draft preparation, LF and NB. Writing—review and editing, all authors. Visualization, LF, NB, and MvK-B. Supervision, NB, MvK-B, and BO. Project administration, NB, MvK-B, and BO. Funding acquisition, NB, MvK-B, and BO. All authors contributed to the article and approved the submitted version.

## Funding

This research was funded by the German Research Foundation (DFG), grant numbers BU 3523/1-1, OH 166/2-1, and KO 3552/8-1. This publication was supported by the DFG and the University of Veterinary Medicine Hannover, Foundation within the funding program Open Access Publishing. The funders had no role in the design of the study; in the collection, analyses, or interpretation of data; in the writing of the manuscript; or in the decision to publish the results.

## Conflict of Interest

Author KS-M is employed by IVD Innovative Veterinary Diagnostics (IVD GmbH), Seelze, Germany.

The remaining authors declare that the research was conducted in the absence of any commercial or financial relationships that could be construed as a potential conflict of interest.

## Publisher’s Note

All claims expressed in this article are solely those of the authors and do not necessarily represent those of their affiliated organizations, or those of the publisher, the editors and the reviewers. Any product that may be evaluated in this article, or claim that may be made by its manufacturer, is not guaranteed or endorsed by the publisher.
