# Supplementary material for: Ex Vivo and In Vitro Analysis Identify a Detrimental Impact of Neutrophil Extracellular Traps on Eye Structures in Equine Recurrent Uveitis
Source: Front Immunol. 2022 Feb 10;13:830871. doi: 10.3389/fimmu.2022.830871 (PMC8896353; doi:10.3389/fimmu.2022.830871)
Supplement: Supplementary file 1 [file DataSheet_1.pdf]

Supplementary Material

Table S1. ERU scoring system (von Borstel *et al.*, 2010), English version adapted from (Kulbrock *et al.*, 2016)

	Score	Iris	Lens	Vitreous	Fundus oculi	Other chronic changes
**healthy**	0	No abnormality detected				
	1	Low-grade depigmentation; focal small synechia	Focal capsular cataract; focal iris residues	Low-grade liquefaction with several filamentous strands of climbed cells and inflammatory products	Single focal chorioretinopathy ("bullet hole lesion")	Low-grade reduction in size of the bulb/reduction in size of the anterior chamber
	2	Low-grade depigmentation; focal large-scale synechia	Multifocal capsular/ subcapsular cataract, local bullous subcapsular/cortical cataract; multifocal iris residues	Low-grade liquefaction; several curtain-like strands of climbed cells and inflammatory products	A few focal chorioretinopathies ("bullet hole lesions"); small area peripapillary chorioretino-pathy ("butterfly lesion")	Moderate reduction in size of the bulb/reduction in size of the anterior chamber; local corneal haze
**ERU**	3	Moderate depigmentation; several focal synechiae	Local reticular capsular/subcapsular cataract; local immature cortical/nuclear cataract	Moderate liquefaction; moderate curtain-like strands of climbed cells and inflammation products, low-grade diffuse yellow haze	Multiple focal chorioretinopathies ("bullet hole lesions"); large-scale peripapillary chorioretinopathy ("butterfly lesion")	Low-grade bulb enlargement/ enlargement of the anterior chamber; laminar corneal haze; low-grade increase in intraocular pressure
	4	Moderate depigmentation; atrophy; circular synechiae	Diffuse immature capsular/subcapsular/ cortical and/or nuclear cataract; lens subluxation	High-grade liquefaction; moderate curtain-like strands of climbed cells and inflammatory products, moderate diffuse yellow haze	Laminar chorioretinopathies/ laminar degeneration of the retina; peripapillary wrinkles of the retina; partial retinal detachment	Moderate bulb enlargement/ enlargement of the anterior chamber; laminar corneal haze/ Haab's Striae; moderate increase in intraocular pressure
	5	High-grade depigmentation; seclusion pupillae	Mature/hypermature cataract; lens luxation	High-grade liquefaction; high-grade visible strands of climbed cells and inflammation products, high-grade diffuse yellow haze	Complete retinal detachment	High-grade bulb enlargement/ enlargement of the anterior chamber; laminar corneal haze/ Haab's Striae; high-grade increase in intraocular pressure; phthisis bulbi

Table S2. Patient data of horses included in screening for NETs. Abbreviation: No. = number; d = days; 1-40 = ERU patients; S1-S5 = control horses with healthy eyes that gave serum samples; V1-V5 = control horses with healthy eyes from whom vitreous body fluid (VBF) samples were derived; OS = left eye; OD = right eye; MAT = microscopic agglutination test; PCR = polymerase chain reaction; G. = *Grippotyphosa*; P. = *Pomona*; I. = *Icterohaemorrhagiae*; C. = *Copenhageni*; Aus. = *Australis*; Aut. = *Autumnalis*; B. = *Bratislava*; S. = *Saxkoebing*; Can. = *Canicola*.

No.	Breed	Age	Coat color	Gender	Affected eye	No. of episodes	Timespan since last episode [d]	ERU score						*Leptospira*		Serovar and titer	Both eyes affected
								Total	Iris	Lens	Vitreous	Fundus	Other changes	MAT	PCR		
1	Oldenburger	7	Chestnut	Gelding	OS	2	21	1	1	0	1	0	1	+	-	G. 1:1600	No
2	German Sport Horse	9	Bay	Gelding	OD			3	0	2	3	0	0	+	-	G. 1:100	Yes
3	Purebred Spanish Horse	15	Bay	Gelding	OS	3	16	5	4	2	5	0	0	+	+	G. 1:3200	No
4	German Riding Pony	12	Bay	Mare	OD		8	3	3	2	3	0	0	+	+	G. 1:800	Yes
5	Westphalian horse	15	Bay	Gelding	OD	2		3	1	3	2	0	2	-	-		No
6	Icelandic horse	11	Black	Gelding	OD	2	23	3	0	3	2	0	0	+	+	G. 1:800, P. 1:3200	No
7	German Sport Horse	16	Bay	Mare	OS	2		3	0	1	3	1	0	+	+	G. 1:800, P. 1:1600	No
8	Westphalian horse	4	Chestnut	Gelding	OD	1	12	4	3	3	4	4	2	+	-	G. 1:100	Yes

No.	Breed	Age	Coat color	Gender	Affected eye	No. of episodes	Timespan since last episode [d]	ERU score						*Leptospira*		Serovar and titer	Both eyes affected
								Total	Iris	Lens	Vitreous	Fundus	Other changes	MAT	PCR		
9	Holsteiner	9	Bay	Gelding	OS	1		2	0	2	1	0	0	+	-	G. 1:100	Yes
10	German Riding Pony	6	Bay	Gelding	OD	2	16	4	0	3	4	2	2	+	+	G. 1:3200, I. 1:200	No
11	Polish Konik	15	Dun	Mare	OD		9	4	0	0	4	0	0	+	+	G. 1:3200, P. 1:3200, C. 1:400, Aus. 1:200, B. 1:100, Aut. 1:400	Yes
12	Haflinger	17	Palomin o	Mare	OS	2	4	3	0	2	3	0	0	+	+	G. 1:3200, P. 1:400	No
13	Hanoverian	10	Chestnut	Mare	OS	2		3	0	3	3	0	2	-	-		No
14	Polish Konik	12	Black	Gelding	OD	2		4	0	1	4	0	0	+	+	G. 1:3200	No
15	Trotter	4	Bay	Mare	OS	2	7	3	0	0	3	0	0	+	+	G. 1:3200, Can. 1:50	No
16	Polish Konik	11	Dun	Gelding	OS	4		3	0	2	3	0	0	-	-		Yes
17	Hanoverian	3	Black	Gelding	OD			1	0	0	1	0	0	-	-		No
18	Warmblood	18	Bay	Mare	OD	2		2	0	2	2	0	0	-	-		Yes
19	Hanoverian	4	Bay	Mare	OD			4	0	1	4	2	0	+	+	G. 1:800	No
20	Hanoverian	7	Bay	Gelding	OD			2	0	2	1	0	0	+	-	G. 1:200	No

No.	Breed	Age	Coat color	Gender	Affected eye	No. of episodes	Timespan since last episode [d]	ERU score						*Leptospira*		Serovar and titer	Both eyes affected
								Total	Iris	Lens	Vitreous	Fundus	Other changes	MAT	PCR		
21	Icelandic horse	6	Bay	Mare	OS			3	0	2	3	0	0	+	-	G. 1:200	No
22	Fjord horse	4	Dun	Gelding	OD	2		2	0	2	0	0	0	+	-	I. 1:100	Yes
23	Westphalian horse	5	Chestnut	Mare	OD			5	0	2	5	0	0	+	+	G. 1:800	Yes
24	Hanoverian	5	Bay	Gelding	OS			2	0	2	1	0	0	+	-	G. 1:100	Yes
25	Oldenburger	4	Chestnut	Gelding	OS			3	0	3	1	0	0	-	-		No
26	Icelandic horse	4	Black	Mare	OD	1	60	3	0	3	1	0	1	+	-	G. 1:50	No
27	Irish Tinker	16	Bay	Gelding	OS			3	0	3	1	1	0	-	-		Yes
28	Trotter	8	Chestnut	Gelding	OS	2		1	0	1	1	0	0	+	-	G. 1:200, I. 1:50	Yes
29	Icelandic horse	7	Dun	Mare	OD		6	4	0	0	4	0	0	+	+	G. 1:3200, P. 1:800, I. 1:100	No
30	Icelandic horse	14	Bay	Gelding	OS			2	0	2	1	0	0	-	-		No
31	Thoroughbred	9	Bay	Gelding	OS	1	7	5	0	3	5	4	1	+	+	G. 1:800, P. 1:1600, Aus. 1:200, B. 1:100, C. 1:400	No

No.	Breed	Age	Coat color	Gender	Affected eye	No. of episodes	Timespan since last episode [d]	ERU score						*Leptospira*		Serovar and titer	Both eyes affected
								Total	Iris	Lens	Vitreous	Fundus	Other changes	MAT	PCR		
32	Icelandic horse	11	Pinto	Gelding	OD	1		2	0	2	1	0	0	+	-	G. 1:100, I. 1:50	No
33	Icelandic horse	15	Chestnut	Mare	OS		1	5	1	5	5	4	4	-	-		Yes
34	Irish Tinker	7	Pinto	Mare	OD	1		1	0	0	1	0	0	+	+	G. 1:3200, P. 1:3200, I. 1:100, Aus. 1:200, B. 1:100, Aut. 1:100, C. 1:200	No
35	Hanoverian	7	Bay	Gelding	OS	3	7	3	0	2	3	0	1	-	+		Yes
36	Icelandic horse	4	Black	Gelding	OS	2	6	2	0	2	1	0	0	+	-	G. 1:50	Yes
37	Hanoverian	4	Bay	Mare	OS			3	0	0	3	0	0	+	+	G. 1:200	Yes
38	German Riding Pony	9	Chestnut	Gelding	OD	1		1	0	0	1	0	0	+	+	S. 1:200	No
39	Icelandic horse	2	Dun	Stallion	OS	1	28	3	0	3	3	0	0	+	+	P. 1:50, Aus. 1:1600, Aut. 1:100, B. 1:400	No
40	German Riding Pony	6	Bay	Mare	OD	2		5	3	3	5	0	4	-	-		Yes

No.	Breed	Age	Coat color	Gender	Affected eye	No. of episodes	Timespan since last episode [d]	ERU score						*Leptospira*		Serovar and titer	Both eyes affected
								**Total**	**Iris**	**Lens**	**Vitreous**	**Fundus**	**Other changes**	**MAT**	**PCR**		
S1	Hanoverian	16	Bay	Stallion	none	0	-	0	0	0	0	0	0	Only serum sample, thus no analysis			No
S2	Hanoverian	22	Bay	Stallion	none	0	-	0	0	0	0	0	0	Only serum sample, thus no analysis			No
S3	Trotter	9	Bay	Mare	none	0	-	0	0	0	0	0	0	Only serum sample, thus no analysis			No
S4	Danish warmblood	18	Bay	Mare	none	0	-	0	0	0	0	0	0	Only serum sample, thus no analysis			No
S5	Hanoverian	11	Bay	Mare	none	0	-	0	0	0	0	0	0	Only serum sample, thus no analysis			No
V1	Hanoverian	17	Black	Mare	none	0	-	Without signs of ERU in pathology report						-	-		No
V2	Hanoverian	17	Black	Mare	none	0	-	Without signs of ERU in pathology report						-	-		No
V3	Oldenburger	13	Gray	Mare	none	0	-	Without signs of ERU in pathology report						-	-		No
V4	Hanoverian	24	Bay	Stallion	none	0	-	Without signs of ERU in pathology report						-	-		No
V5	Arabian half-breed	25	Gray	Mare	none	0	-	Without signs of ERU in pathology report						-	-		No

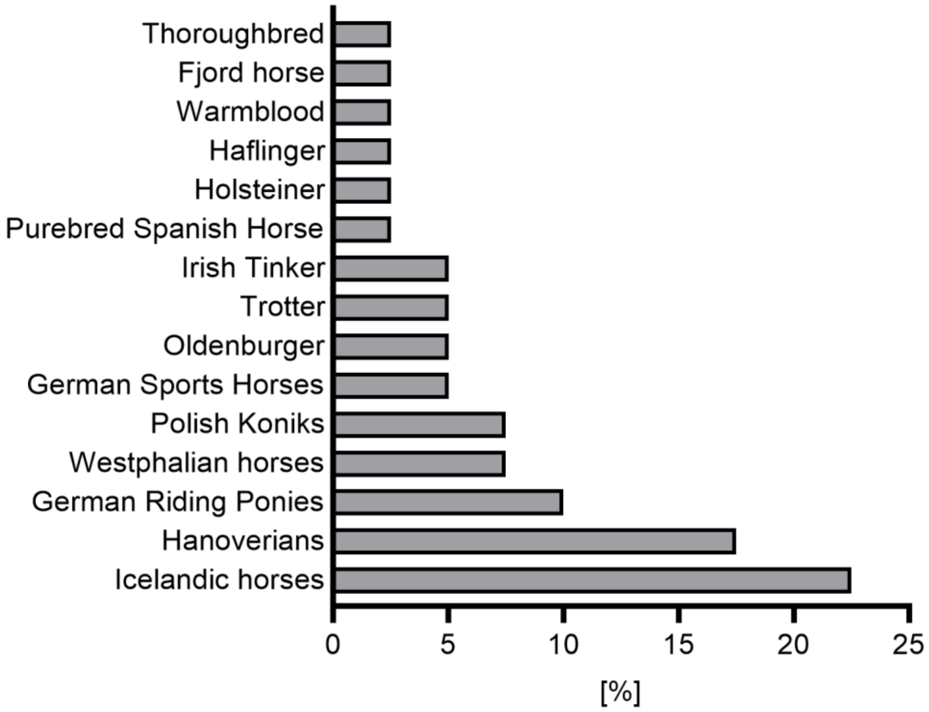

Fig. S1. Breed distribution of horses included in screening for NETs. The proportion in percent of each breed included in the NET analysis is depicted.

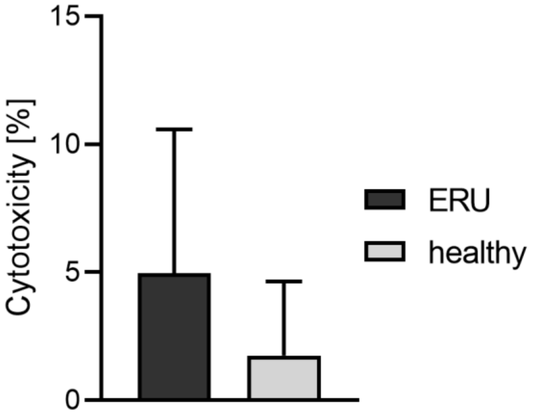

Fig. S2. Lactate dehydrogenase (LDH) values of equine VBF. LDH values were determined in the VBF samples included in the cell culture experiment (see Fig. 4), comparing samples of healthy with ERU-diseased horses. A one-tailed unpaired Student's t-test revealed no significant difference (*p*=0.14).

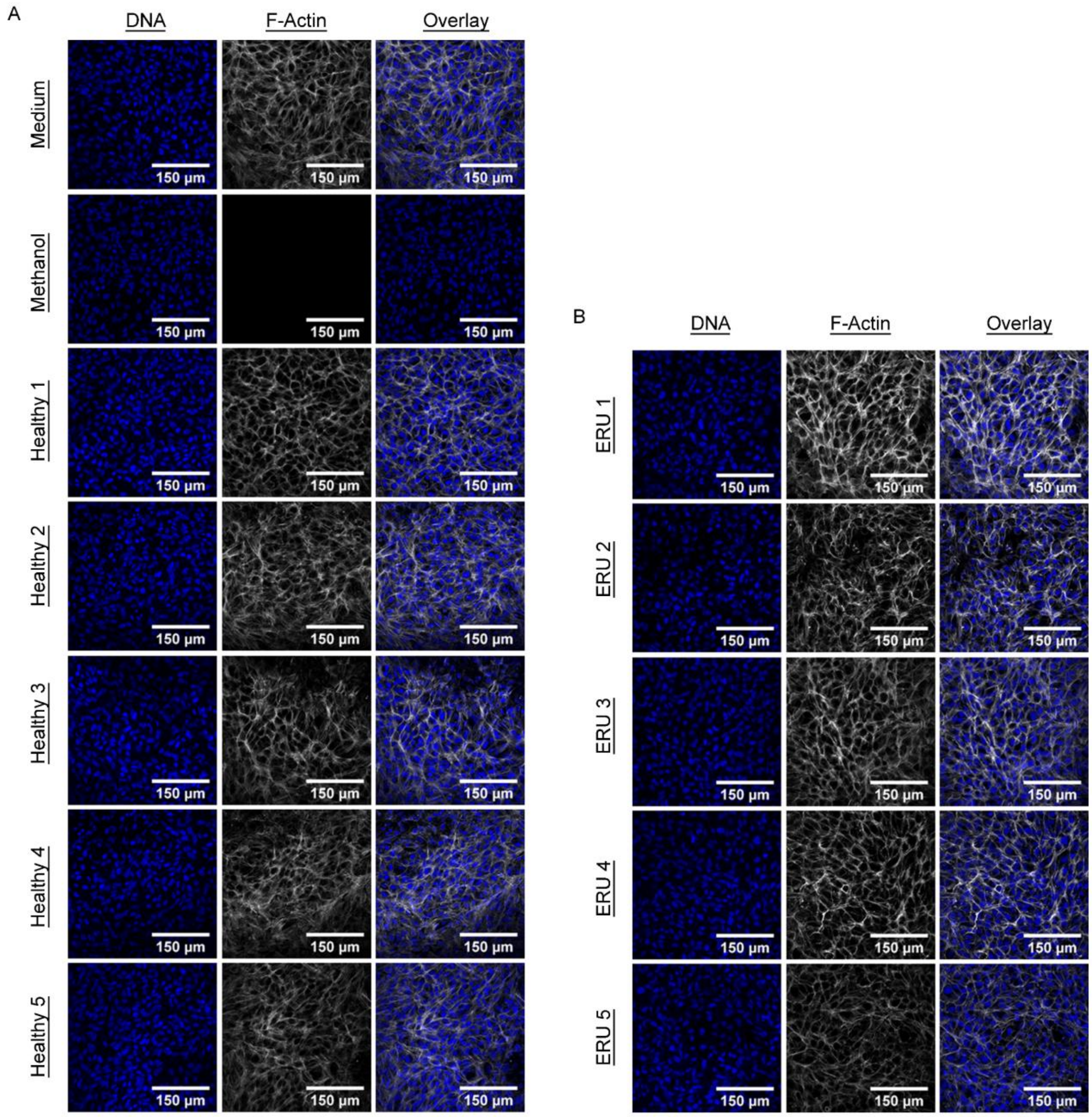

Fig. S3. Interaction of equine VBF with cells of the outer blood-retina barrier. Confluent ARPE-19 cells were exposed to VBF of five horses with healthy eyes and five ERU-diseased horses. One representative image of the cells per stimulus, including the incubation with medium (DMEM:F12, 1% Penicillin-Streptomycin), methanol (final 70%), VBF of horses with healthy eyes and VBF derived from ERU patients, is depicted. Confocal microscopy was performed after immunofluorescence staining. Single channel pictures and overlays are presented. a) controls and healthy horses, b) ERU horses.

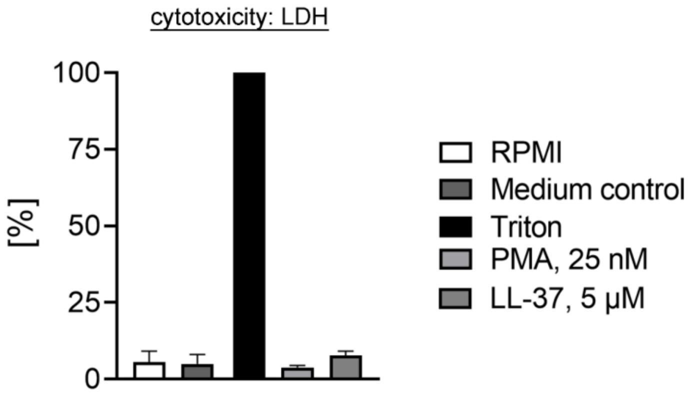

Fig. S4. Interaction of NET inducing substances with retinal pigment epithelium (RPE). Confluent ARPE-19 cells were exposed to NET inducing substances (n=1), either phorbol myristate acetate (PMA) or the human cathelicidin LL-37. Cytotoxicity was measured by the amount of released LDH. PMA and LL-37 did not result in remarkable changes of LDH release. The bars represent the mean ± SD out of two different wells per stimulus.
